# Characterization of a multicenter pediatric-hydrocephalus shunt biobank

**DOI:** 10.1186/s12987-020-00211-6

**Published:** 2020-07-18

**Authors:** Jacob Gluski, Paul Zajciw, Prashant Hariharan, Amanda Morgan, Diego M. Morales, Andrew Jea, William Whitehead, Neena Marupudi, Steven Ham, Sandeep Sood, James P. McAllister, David D. Limbrick, Carolyn A. Harris

**Affiliations:** 1 Wayne State University School of Medicine, 540 E. Canfield Avenue, Detroit, MI 48201 USA; 2 Wayne State University Dept. of Chemical Engineering and Materials Science, 6135 Woodward Avenue, Rm 1413, Detroit, MI 48202 USA; 3 Washington University School of Medicine Dept. of Neurological Surgery, 660 S. Euclid Avenue, St. Louis, MO 63110 USA; 4 Riley Hospital for Children at IU Health, 705 Riley Hospital Drive, Indianapolis, IN 46202 USA; 5 Texas Children’s Hospital, Baylor College of Medicine, 6701 Fannin Street, Suite 1230.01, Houston, TX 77030 USA; 6 Children’s Hospital of Michigan Dept. of Neurosurgery, 3901 Beaubien Boulevard, 2nd Floor Carl’s Building, Detroit, MI 48201 USA

**Keywords:** Hydrocephalus, Biobank, Shunt failure, Shunt obstruction, CSF = cerebrospinal fluid, Ventriculoperitoneal shunt, Retrospective cohort, Translational, Multicenter, Improving surgical outcomes

## Abstract

**Background:**

Pediatric hydrocephalus is a devastating and costly disease. The mainstay of treatment is still surgical shunting of cerebrospinal fluid (CSF). These shunts fail at a high rate and impose a significant burden on patients, their families and society. The relationship between clinical decision making and shunt failure is poorly understood and multifaceted, but catheter occlusion remains the most frequent cause of shunt complications. In order to investigate factors that affect shunt failure, we have established the Wayne State University (WSU) shunt biobank.

**Methods:**

To date, four hospital centers have contributed various components of failed shunts and CSF from patients diagnosed with hydrocephalus before adulthood. The hardware samples are transported in paraformaldehyde and transferred to phosphate-buffered saline with sodium azide upon deposit into the biobank. Once in the bank, they are then available for study. Informed consent is obtained by the local center before corresponding clinical data are entered into a REDCap database. Data such as hydrocephalus etiology and details of shunt revision history. All data are entered under a coded identifier.

**Results:**

293 shunt samples were collected from 228 pediatric patients starting from May 2015 to September 2019. We saw a significant difference in the number of revisions per patient between centers (Kruskal–Wallis H test, *p* value < 0.001). The leading etiology at all centers was post-hemorrhagic hydrocephalus, a fisher’s exact test showed there to be statistically significant differences in etiology between center (p = 0.01). Regression showed age (p < 0.01), race (p = 0.038) and hospital-center (p < 0.001) to explain significant variance in the number of revisions. Our model accounted for 31.9% of the variance in revisions. Generalized linear modeling showed hydrocephalus etiology (p < 0.001), age (p < 0.001), weight and physician (p < 0.001) to impact the number of ventricular obstructions.

**Conclusion:**

The retrospective analysis identified that differences exist between currently enrolled centers, although further work is needed before clinically actionable recommendations can be made. Moreover, the variables collected from this chart review explain a meaningful amount of variance in the number of revision surgeries. Future work will expand on the contribution of different site-specific and patient-specific factors to identify potential cause and effect relationships.

## Background

Pediatric hydrocephalus, a condition caused by altered cerebrospinal fluid (CSF dynamics), affects approximately 1 in 1100 people in the USA [1]. The perturbation of CSF homeostasis can lead to increased ventricular size and compression of vital brain structures [2]. There are a variety of hydrocephalus etiologies. Those most common in pediatrics include congenital central nervous system (CNS) malformations, infection, intraventricular hemorrhage (IVH), genetic defects, trauma, and teratogens [3]. Risk factors associated with pediatric hydrocephalus include birth weight less than 1500 grams, prematurity (gestational age less than or equal to 30 weeks), maternal diabetes, low socioeconomic status, and male sex. Incidence is lower in Asians than other races [4, 5].

Shunting of CSF from the ventricles became the mainstay of treatment for pediatric hydrocephalus in the 1950s, with ventriculoatrial shunts (VAS) being the preferred configuration. Shunts utilizing valves for CSF pressure or flow control soon became the norm. In the 1980s the VAS was superseded by the ventriculoperitoneal shunt (VPS) for hydrocephalus management. In the 1990s endoscopic third ventriculostomy (ETV) became an option to manage some types of obstructive hydrocephalus, obviating the need for fallible shunt hardware [6]. In 2005 and again in 2012, the National Institutes of Health sponsored an expert panel to discuss priorities for hydrocephalus research, this panel concluded both times that current methods of diagnosis, treatment and outcome monitoring need improvement [7, 8]. More recently, the Hydrocephalus Clinical Research Network (HCRN), a consortium of 14 North American Pediatric Hospitals, developed a standardized operating protocol that was shown to reduce rates of post-operative infection associated with shunt procedures [9].

Pediatric CSF shunt systems have a failure rate of up to 85% within 10 years from initial insertion [10, 11]. Annual hospitalizations for hydrocephalus have reached 70,000 per year in the USA. Nearly all patients with hydrocephalus (98%) will experience shunt failure in their lifetime [12]. Pediatric patients experience higher rates of failure, with 40% of shunts failing within 2 years of implantation [13]. The annual cost of pediatric hydrocephalus intervention is approximately $195.5–204.5 million [14] and the overall burden to the healthcare system is between $1.4 and 2.0 billion; over half of these expenses are due to shunt revisions [15].

Tissue obstruction of the proximal (i.e. ventricular) catheter is the main source of failure in VPS systems, accounting for approximately 50% of failures within the pediatric population [16]. The mechanisms underlying this failure are still poorly understood. Sekhar et al. [17] provided the earliest description of the cell types involved in shunt catheter occlusion, and more recent efforts have shown that astrocytes and microglia likely play a central role in this tissue obstruction [18]. The molecular pathways underpinning this phenomenon, which could serve as targets for pharmacologic intervention, are not yet known. Likewise, there is a lack of understanding as to how clinical decision-making influences shunt failure rates. With the new opportunities offered by cheaper sequencing and tissue-clarification, the field stands poised to gain a deeper understanding of the biological processes underlying shunt failure due to obstruction. To facilitate investigation of this question, we created a national biorepository of all failed shunt hardware, following other institutions that have created biobanks for different medical conditions [19, 20]. Centered at Wayne State University (WSU), this shunt biobank and corresponding clinical database has the potential to be a global cohort of explanted central nervous system hardware.

In this paper, the authors set out to detail the biobank and demonstrate how participating centers can benchmark their performance against others. Moreover, by modeling the effects of collected variables on the number of revisions, this paper attempts to build the foundation for prognostic algorithms—something which has been lacking for pediatric hydrocephalus.

## Materials and methods

### Ethics approval and study population

Written informed consent was obtained from all patients or their legally authorized representative. The patient population includes a vulnerable group (children), but the study is aimed at addressing the health needs of this group and cannot be conducted in a non-vulnerable group. The biobank has samples from individuals who were aged between 36 days and 42 years, with a mean of 9.23 years (SD = 8.39). Samples were collected from individuals with any hydrocephalus etiology except normal pressure hydrocephalus and with any clinical history. Patients were evaluated by local centers according to their individual guidelines, and samples were only collected if the shunt malfunction indicated surgical intervention.

### Current centers

Children’s Hospital of Michigan and Wayne State University (WSU), St. Louis Children’s Hospital and Washington University School of Medicine in St. Louis (WUSM), Texas Children’s Hospital -Baylor College of Medicine (TEX), Riley Children’s Hospital—Indiana University Health (RC), the Children’s Hospital of Alabama at University of Alabama Birmingham (ALA), and John’s Hopkins Medicine (JHU). ALA and JHU had not contributed samples at the time of analysis.

### Sample collection

After removal by a surgeon, the shunt samples were placed in a solution of sterile 4% (w/v) paraformaldehyde (PFA). They were then given a unique identifier and deidentified to those who performed the analyses. Samples were shipped to the coordinating center at room temperature. Upon arrival, the shunt components were changed to a solution of 1X PBS with 0.01% (w/v) Sodium Azide and stored at 4 °C. The solution was refreshed monthly. For the samples associated with CSF, this was collected intraoperatively: most commonly during final testing of the shunt dynamics. If CSF was collected, the time elapsed between collection and processing was noted. The CSF was kept below 4 °C until it was spun down at 1000 g for 6 min. The supernatant was then aliquoted into 1.5 mL Eppendorf polypropylene microcentrifuge tubes. The supernatant was stored at − 80 °C and the cell pellet was stored in liquid nitrogen.

Once a patient is enrolled in the study, a review of all their operative reports is performed in order to gather their history of shunt revisions. Some of the clinical variables collected were hydrocephalus etiology, demographics, suspected cause for hardware removal, physician performing procedure, shunt configuration, whether the catheter was adherent to a ventricular wall or the choroid plexus, number of prior revisions, number of ventricular catheters, and number of ventricular catheter obstructions. New variables were created in REDCap using the subtraction of dates; for example, length of hardware implantation = date of surgery – date of hardware implantation. Additional file 1: Table S1 shows all the variables collected. Hydrocephalus etiology was determined by the pre-existing protocols practiced by each center’s neurosurgeons and neuroradiologists.

In order to determine our collection rate, the total number of procedures performed at each center during the dates of collection were obtained from de-identified departmental records.

### Statistical analysis

SPSS for Windows version 25.0 was used. The Chi square test was used to check for differences in race between patients and census data for the metropolitan areas where our centers are located. Fisher’s exact test was used to check for differences in hydrocephalus etiologies between centers. The Kruskal–Wallis test was used to determine if significant differences existed between study groups. Dunn’s post hoc test was used for pairwise comparisons. Hierarchical linear regression was used to determine the amount of variance explained by variables on the total number of revisions. The dependent variables were made ready for analysis by a square root transformation. Only the first sample collected from patients was used in regression analysis. Residuals were plotted to assess normality and Cook’s distance was used to check for cases that disproportionately skewed the model. Effects in the models were checked for collinearity. Generalized Linear modeling was used to model the effects of similar variables on the number of ventricular obstructions, the residuals and Cook’s distance were checked to validate the model.

### Clinical database

Study data were collected and managed using REDCap electronic data capture tools hosted at Wayne State University [21, 22]. REDCap (Research Electronic Data Capture) is a secure, web-based software platform designed to support data capture for research studies. Each participating center is responsible for the collection and maintenance of their data. All centers can access the entirety of the clinical data in our REDCap database.

## Results

### Current biobank content

Across these 4 centers, to date we have enrolled 228 pediatric-hydrocephalus patients, from whom 293 samples have been collected (Table 1); the majority come from WSU and WUSM and most (75.4%) included a proximal catheter. The collection rates (Table 1) vary between centers and from year to year, with a total colleciton rate of 21%. Records for the total number of revision procedures at WUSM were missing from 2015 and incomplete in 2016.

**Table 1:** Current biobank content

	Center				
	WSU	WUSM	TEX	RC	Total
Number of patients	73	109	34	12	228
Number of samples	113	132	34	14	293
Number of samples associated with CSF	40	36	–	–	76
Mean samples per patient	1.58	1.21	1.00	1.17	1.29
Sample breakdown by hardware type					
Samples which include a ventricular catheter	101	81	28	11	221
Samples which include a valve	2	88	5	1	96
Samples which include a peritoneal catheter	2	33	4	1	40
Samples which include an EVD	5	1	–	–	6
Samples which include a subdural catheter	3	5	–	–	8
Samples which include a reservoir	1	14	–	1	16
Number of samples per year					
2015	–	24	–	–	24
2016	49	15	–	–	64
2017	32	27	1	–	60
2018	28	37	23	1	89
2019	5	29	10	12	56
Collection rates as a percentage of total revision surgeries performed					
2015	–	Missing	–	–	–
2016	31%	Missing	–	–	–
2017	15%	42%	3%	–	20%
2018	19%	43%	15%	33%	22%
2019	7%	46%	7%	28%	18%

The character “–” denotes a cell whose value is zero *CSF* cerebrospinal fluid *EVD* external ventricular drain

The demographics of the patients already enrolled (Table 2) show a prevalence of males; however, this was not statistically significant. The total percentage of African American patients was significantly higher compared to the general population (Chi square p = 0.0013); however, this significance disappears when controlling for the percentage of African Americans in the metropolitan areas our hospitals serve (Chi square p = 0.8278). Patient age at sample collection was significantly different between the sites (Kruskal–Wallis H test p < 0.001).

**Table 2:**
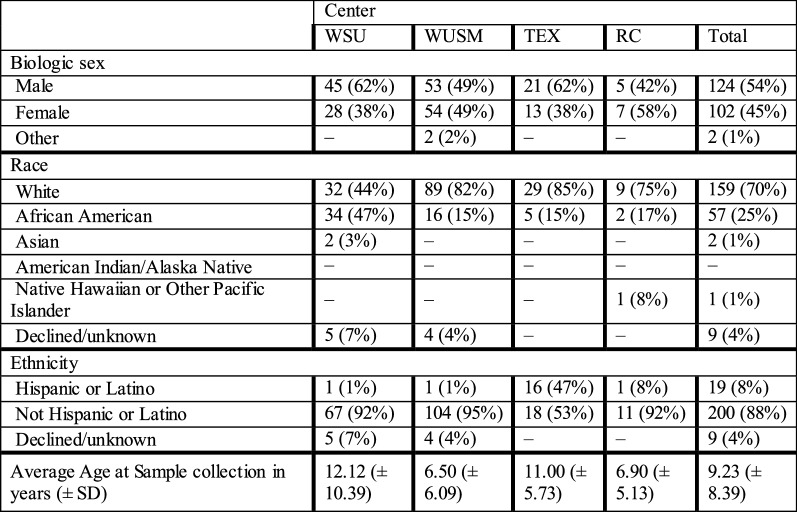
Demographics of patients with samples in the biobank

Data represent counts of patients unless otherwise denoted. Percentages were calculated down the column for each variable, illustrated with the darker cell borders. The character “–”represents a cell whose value is zero, *SD* standard deviation

### Hydrocephalus history

The hydrocephalus etiologies (Table 3) of patients in our biobank varied significantly between centers (Fisher’s Exact Test p = 0.01); however, the leading etiology at all centers was intraventricular hemorrhage of prematurity.

**Table 3:**
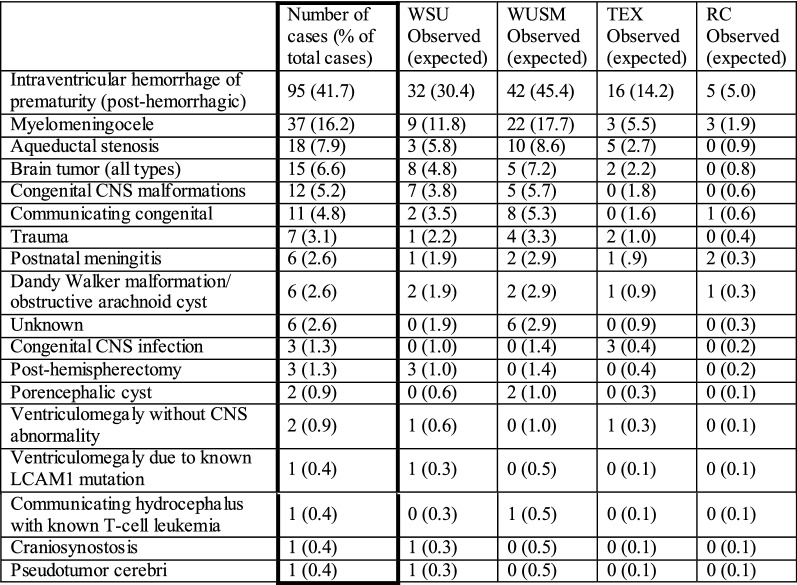
Hydrocephalus etiology

*Observed* refers to the counts of patients with each etiology. *Expected* refers to the expected frequency of each etiology if there were not differences between the centers *CNS* central nervous system *LCAM1* L1 cell adhesion molecule

The most commonly suspected cause for hardware removal (Table 4) was obstruction of the proximal catheter, with 41.2% of samples having it as the sole suspected cause of failure. One notable finding was that while valve obstruction and disconnection each accounted for a small number of samples where they were the sole cause for removal (3.4% and 2.4% respectively); however, both were commonly included when multiple causes of failure were suspected.

**Table 4:** Suspected cause for hardware removal

	Number of samples with only one suspected cause of failure (% of total samples)	Number of samples, including multiple suspected causes (% of total samples)
Proximal catheter obstruction	121 (41.2)	148 (50.5)
Valve obstruction	10 (3.4)	31 (10.6)
Multiple suspected causes	37 (12.6)	NA
Externalization due to infection	26 (8.9)	29 (9.9)
Internalization to remove EVD	20 (6.8)	20 (6.8)
Distal catheter obstruction	9 (3.1)	15 (5.1)
Disconnection	7 (2.4)	15 (5.1)
Switching shunt configuration	11 (3.8)	11 (3.8)
Removal of original reservoir	10 (3.4)	10 (3.4)
Over-drainage	6 (2.0)	8 (2.7)
Reservoir malfunction	4 (1.4)	6 (2.0)
Truncated catheter	4 (1.4)	4 (1.4)
Unknown	4 (1.4)	4 (1.4)
Upgrading valve	4 (1.4)	4 (1.4)
Fracture of proximal catheter	2 (0.7)	4 (1.4)
No longer shunt dependent	4 (1.4)	4 (1.4)
Fracture of distal catheter	2 (0.7)	3 (1.0)
Externalized due to other cause	2 (0.7)	3 (1.0)
Ventriculomegaly not otherwise specified	3 (1.0)	3 (1.0)
Wound dehiscence	3 (1.0)	3 (1.0)
Externalization due to pseudocyst	2 (0.7)	2 (0.7)
Successful ETV	1 (0.3)	1 (0.3)
Pseudo-meningocele formed around valve	1 (0.3)	1 (0.3)

The counts of samples with each suspected cause of failure are shown. The first column displays *Multiple suspected causes* as its own category, while in the second column this category has been broken into component causes. (% of total samples) = (n of cause)/(293) *ETV* endoscopic third ventriculostomy *EVD* external ventricular drain *NA* not applicable

When the indication for failure included infection, we cross referenced lab results to check if the patient had a positive CSF culture during their admission. Out of the 29 samples that were removed for suspected infection: 6 had negative CSF cultures, 20 had positive cultures and no cultures were ever obtained for 3. Additionally, 4 others in whom infection was not suspected pre-operatively showed positive CSF cultures.

**Fig. 1:**
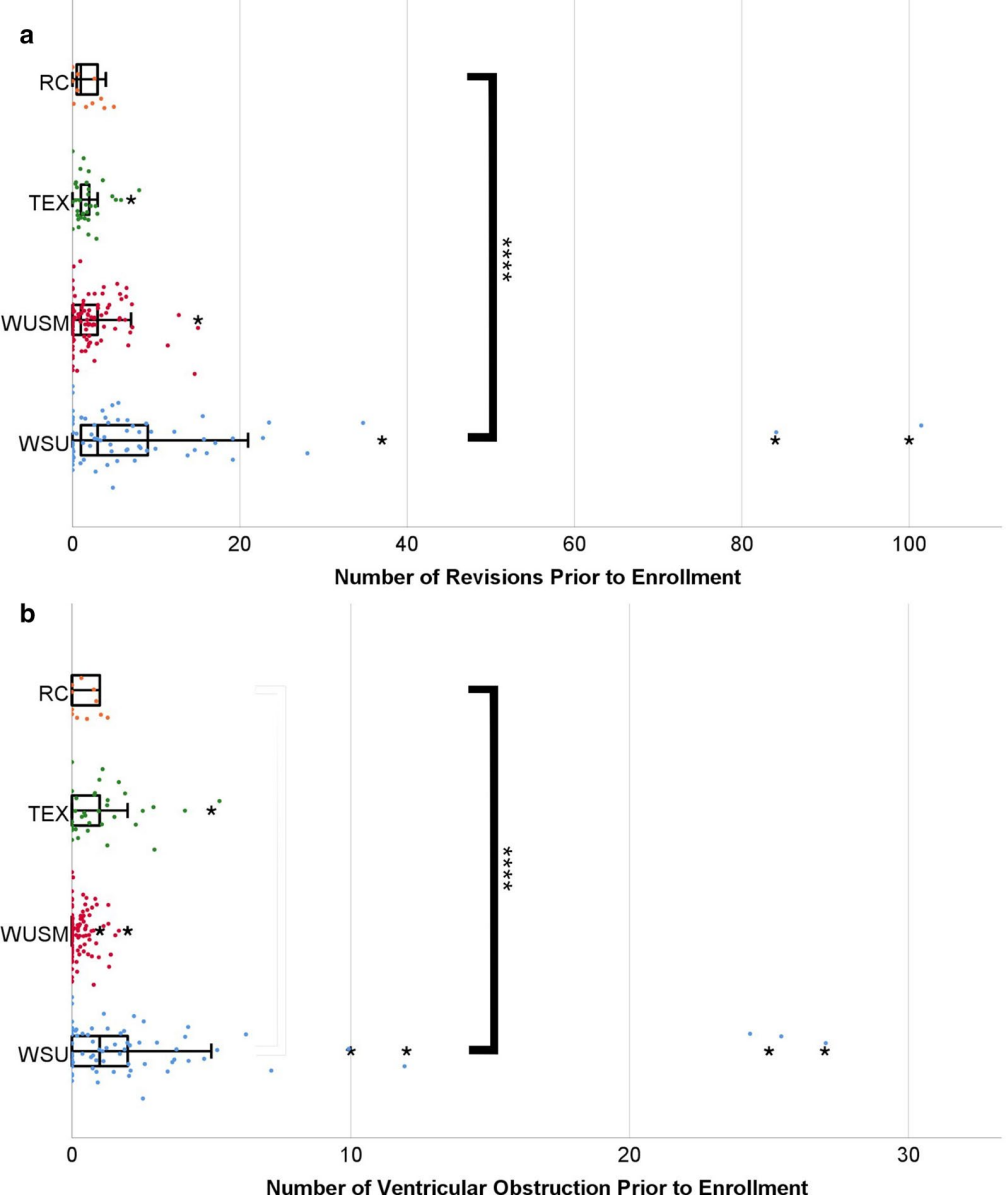
Patient History of Revisions and Ventricular Obstructions Reveals Historic Differences Between Sites. **a** The mean number of prior revisions are as follows: WSU 8.53, WUSM 1.99, TEX 1.65, and RC 1.64. **b** The mean number of prior ventricular obstructions are as follows: WSU 2.73, WUSM 0.15, TEX 1.03, and RC 0.36. **** p-value < 0.0001 by Kruskal–Wallis H test. *denotes numeric outliers more than 3 SDs away from the mean for each center

**Fig. 2:**
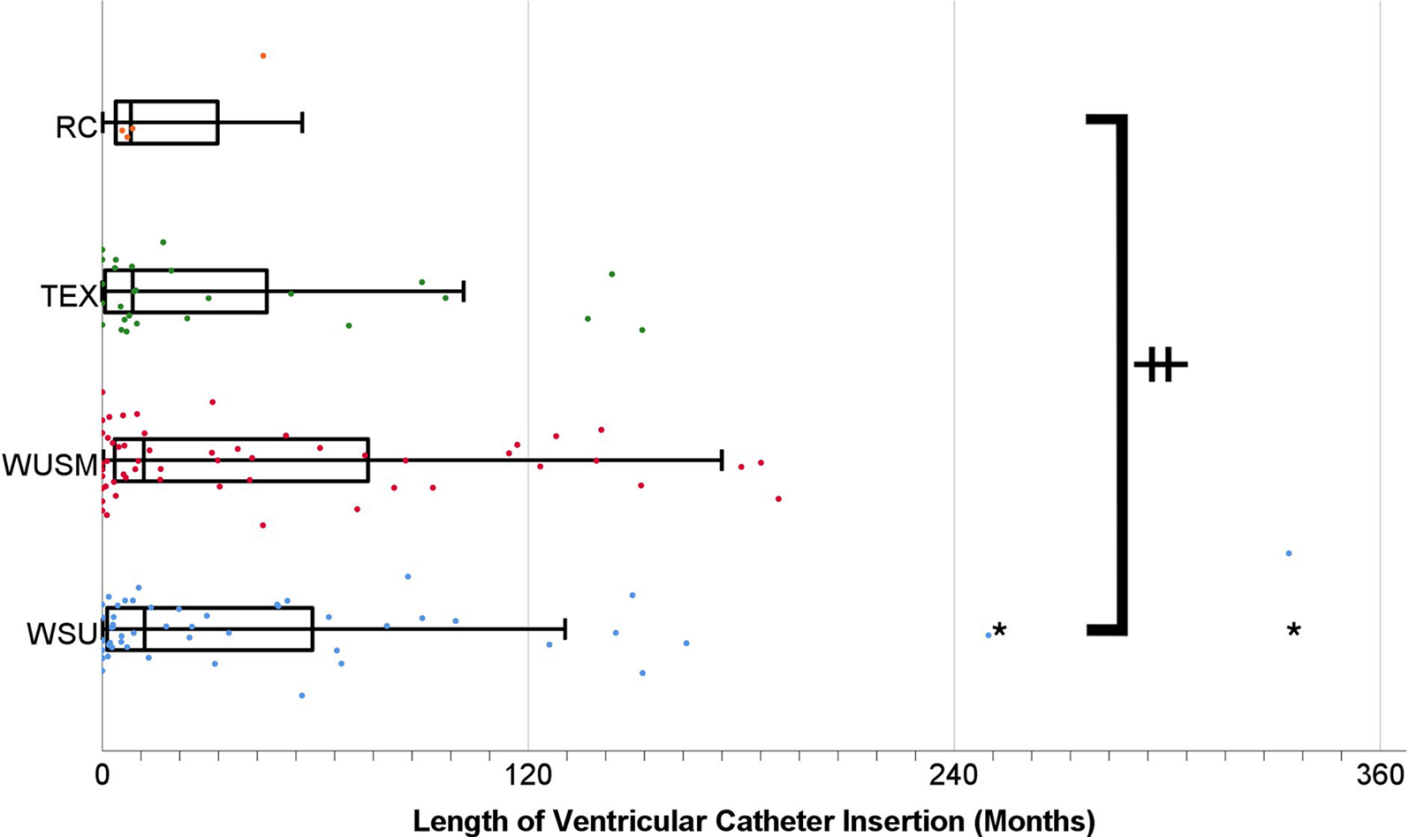
Similar Duration of Ventricular Catheter Implant Between Sites Shows Recent Performance Is More Similar Than Revision History Suggests. Box and whisker plots showing median and interquartile range overlay scatterplots of the duration each ventricular catheter sample was implanted. Data is stratified by site and displayed in months. ǂ indicates p = 0.609. *denotes numeric outliers more than 3 SDs away from the mean for each center

The number of revisions prior to patient enrollment in the biobank (Fig. 1a) differed significantly between centers (Kruskal–Wallis H test p < 0.001); the medians (and interquartile ranges) are as follows: WSU 3 (8), WUSM 1 (3), TEX 1 (1), and RC 1 (4). Pairwise comparisons (Dunn’s post hoc test) showed WSU to be significantly higher than TEX and WUSM (p = 0.003 and p < 0.001 respectively). All other comparisons were not significant. The number of ventricular catheter obstructions prior to enrollment (Fig. 1b) was also significantly different between centers (Kruskal–Wallis H test p < 0.001). Pairwise comparison (Dunn’s post hoc test) showed WUSM to be significantly lower than TEX and WSU (p < 0.001 and p < 0.001 respectively), all other comparisons were not significant. One other metric by which centers can be compared is the mean length of time that each ventricular catheter was implanted before failing (Fig. 2). The median lengths of insertion in months (and interquartile ranges) were as follows: WSU 5.84 (52.08), WUSM 8.97 (64.54), TEX 8.61 (55.16), and RC 8.01 (42.48). There was not a significant difference between the centers (Kruskal–Wallis H test p = 0.609).

Hierarchical linear regression was performed to see if site, and other variables, significantly impacted the number of revisions prior to enrollment when controlling for sex, age at surgery, race, ethnicity, and weight. The R^2^ of these controlled variables was .226, with age having significant (p < 0.001) positive correlation. Race also significantly (p = 0.038) affected the number of revisions; the only significant pairwise comparison (Dunn’s post hoc p = 0.04) showed African Americans to have had more revisions than Caucasians. No other control variables reached significance. The predictor variables included in the model were site, shunt configuration (VPS, VAS, LPS etc.), hydrocephalus etiology, reason for removal, and physician performing the operation. The total R^2^ of the model was .319, thus the R^2^ change due to the predictor variables was .093 with site having a significant effect (p < 0.001). Notably, hydrocephalus etiology (p = 0.493), shunt configuration (p = 0.070), and physician (p = 0.706) did not reach significance.

Similar modeling was not valid when performed on the number of ventricular obstructions due to the non-normality of the residual plots (data not shown), therefore generalized linear modeling was used. Hydrocephalus Etiology (p < 0.001), physician performing the operation (p < 0.001), patient weight at admission (p = 0.004) and age (p < 0.001) had significant effects. When examining the parameter effects for hydrocephalus etiology post-hemorrhagic (p < 0.001), myelomeningocele (p < 0.001), aqueductal stenosis (p = 0.011), Dandy-Walker malformation (p < 0.001), congenital CNS malformations (p = 0.017), unknown (p = 0.001), and other (p = 0.023) had positive correlations with ventricular obstruction, the largest of which was Dandy-Walker malformation. No individual physician reached significance. The effect of weight in the model showed a negative correlation with the number of ventricular obstructions, while age showed a positive correlation.

## Discussion

This biobank has a broad range of sample types from pediatric hydrocephalus patients with various etiologies; as such, it allows for easy investigation into the prevalence of different etiologies and reasons for failure. As it stands, our bank shows a lower prevalence of hydrocephalus due to brain tumors than previously reported [18]. The prevalence across the various causes of failure was similar to previously reported values [18]. The rate of failure due to suspected infection was similar to historic rates, but not as low as during the HCRN study (5.7%) [9]. This is understandable as sample collection for this study was already underway when that protocol was published, and its implementation would take some time for centers to adopt. Additionally, our study shows a persistence of intraventricular hemorrhage of prematurity as the leading cause of hydrocephalus, despite recent reductions in the rates of IVH [23, 24]. The most common etiology at all centers was post-hemorrhagic hydrocephalus. A final advantage over previous collections is the multicenter design, which will increase the generalizability of future studies and allow for comparisons between centers. The diversity and generalizability of this biobank are unique features that increase its utility to fellow researchers.

Significant differences existed between centers for the primary outcomes of number of revisions and proximal catheter obstructions. Regression showed non-modifiable demographic factors and clinical site to predict revision number. Additionally, hydrocephalus etiology, physician performing the surgery, weight, and age predicted variance in the number of ventricular obstructions. While this is not immediately actionable in a clinical setting, it does provide prognostic information.

The historic measurements of performance, i.e. prior revisions and ventricular obstructions, showed a difference between sites; however, duration of implantation for ventricular catheters did not. Since the latter reflects the most recent surgeries, this implies that the clinical sites are currently performing more similarly than in the past.

No other study has attempted to build a prognostic algorithm for all etiologies of pediatric hydrocephalus. The lack of prior work in this area is likely due to the heterogeneity between patients. The number of samples currently in the bank is not yet sufficient to deal with this heterogeneity, as shown by the R^2^ = 0.319. Since we only modeled the effect of the most commonly studied variables, this insufficiency is evidence that a broad approach will be required to understand these complex relationships. The initial reason why we elected for a broad approach was recent studies showing microglia and astrocytes to compose most of the tissue obstructing proximal catheters and previous collections of failed shunts did not have variables relating to inflammation [18, 25]. By casting a wide net during the retrospective review, this biobank can better serve future studies.

The extensive characterization of revision history is a component of this broad focus, it is our hope that this will allow future studies to characterize the biologic impact of long-term clinical decision making. Moreover, by collecting failed shunt samples longitudinally, we have several patients for whom multiple samples are banked. This will allow for intra-patient comparisons during translational studies. For example, studying how immune responses adapt to repeated introduction of foreign material in the CNS.

We invite readers at other hospitals to join as one of our centers and contribute samples to the biobank. Additionally, we welcome new collaborators to make use of the current biobank.

We have begun our own ex vivo studies using some samples from the biobank to better understand proximal obstruction. As a part of our analysis, we are recording the degree of flow volume transport with a buffered solution column. This, along with cellular imaging, provides a detailed and objective assessment of ventricular obstruction. We have found that physical obstruction is not always present when an operative report lists ventricular obstruction as the suspected cause for removal. This disparity could explain why physician performing the operation was predictive of the number ventricular obstructions. By better understanding the adherent cells, new materials and coatings can be trialed to better repel them.

The major limitation to our current study was our collection rate. The low collection rate leaves room for sampling bias which could have affected the outcomes reported in this study. Specific information such as hydrocephalus etiology or revision history could not be obtained for samples not collected since those patients had not enrolled in the study. Qualitatively, we observed that a high percentage of samples not collected were distal catheters which had fractured and valves which were obstructed. Therefore, Table 4 likely underestimates the true prevalence for these causes. This bias could be explained by the prominence of proximal obstruction in current literature, lending surgeons to more often remember to save proximal catheters. In general, there are two major hurdles for a center in obtaining a high collection rate. The first is working with surgeons to adopt a new research protocol and collect failed shunts. The other issue is timely communication to the research team so that consent can be obtained before the family leaves. This second issue can be a major hurdle due to emergent nature of shunt failure.

There is another factor which could affect collection rates and contribute to unintentional selection bias: shunts that are clinically found to be obstructed, or otherwise have failed, but are adherent to underlying tissue and are abandoned in the patient. In our experience, this represents a very small number of proximal catheters. Ultimately, we defer to clinical judgement and see this as a non-modifiable factor until further advances in catheter material decrease rates of tissue adherence.

In the future, there are two changes which we would like to institute. The first is to use pre-operative imaging to determine when a proximal catheter is in contact with choroid plexus vs a ventricular wall, instead of relying on the operative report. Understanding this correlation may lead to new investigative strategies to mechanisms of shunt obstruction. The second change is to include additional variables related to long-term medications and comorbid conditions. Future uses of the clinical data will take into consideration the finding from this study that clinical site should be controlled for during regression analyses until the underlying cause and effect relationships are better understood; additionally, further work is needed to elucidate why revisions differed between sites.

## Conclusion

We have created a biobank for samples from failed shunt systems in pediatric patients with hydrocephalus for which there is a corresponding database with clinical variables. Currently 6 centers are participating; however, only 4 were presented in this paper due to the limited number of samples from 2 newer centers. Among the 4 centers, there were significant differences in patient age and the number of revisions prior to enrollment in our study; however, the mean interval between replacement of ventricular catheters did not vary significantly. Our current model accounted for 31.9% of the variability in the total number of revisions. As our N increases, we will be able to add more variables to our model and hopefully account for a larger amount of variance. The ultimate goal is a prognostic algorithm.

## Supplementary information

**Additional file 1: Table S1.** Variables collected from electronic medical record.

## Data Availability

The datasets analyzed during the current study are not publicly available due to privacy concerns surrounding HIPPA but are available from the corresponding author on reasonable request.

## Abbreviations

WSU: Wayne State University
CSF: Cerebrospinal fluid
PFA: Paraformaldehyde
CNS: Central nervous system
IVH: Intraventricular hemorrhage
VAS: Ventriculoartial shunt
VPS: Ventriculoperitoneal shunt
ETV: Endoscopic third ventriculostomy
HCRN: Hydrocephalus Clinical Research Network
NIH: National institutes of health
IRB: Institutional review board
EVD: External ventricular drain
ICP: Intracranial pressure
REDCap: Research Electronic Data Capture
MTA: Material transfer agreement
WUSM: Washington University of St. Louis, School of Medicine
TEX: Texas Children’s Hospital, Baylor College of Medicine
RC: Riley Children’s Hospital, Indiana University Health
ALA: Children’s Hospital of Alabama at University of Alabama Birmingham
JHU: John’s Hopkins Medicine
SD: Standard deviation
