# Supplementary material for: Characterization of a multicenter pediatric-hydrocephalus shunt biobank
Source: Fluids Barriers CNS. 2020 Jul 18;17:45. doi: 10.1186/s12987-020-00211-6 (PMC7368709; doi:10.1186/s12987-020-00211-6)
Supplement: Supplementary file 1 — Additional file 1: Table S1. Variables collected from electronic medical record. [file 12987_2020_211_MOESM1_ESM.docx]

| Table S1:  REDCap Variable | Possible Entries |
| --- | --- |
| Patient ID | Unique ID |
| Hydrocephalous Etiology | Intraventricular hemorrhage of prematurity (post-hemorrhagic hydrocephalus), brain tumor (all types), myelomeningocele, aqueductal stenosis, trauma, Dandy Walker malformation/obstructive arachnoid cyst, post-natal meningitis, congenital CNS infection, craniosynostosis, pseudotumor cerebri, macrocephaly/ventriculomegaly without CNS abnormality, congenital CNS malformations, communicating congenital hydrocephalus, unknown, other (enter manually) |
| Transfer patient | Yes, no, unknown, other |
| Patient site | WSU, JHU, WUSM, TEX, ALA, RC |
| Date of birth | Any date/unknown |
| Sex | Male, female, unknown, other |
| race | White, African American, Asian, American Indian/Alaska Native, Native Hawaiian or Other Pacific Islander, declined/unknown |
| Ethnicity | Hispanic or Latino, not Hispanic or Latino, Declined/Unknown |
| Sample ID | Unique ID |
| Date of Banking | Any date/unknown |
| CSF associated with sample | Yes, no, unknown, other |
| CSF sample ID | Unique ID |
| Volume of CSF | Any number in μL, mL |
| CSF banked within | 24 hours, 48 hours, 72 hours, other |
| History of ETV | Yes, no, other |
| Date of ETV | Any date/unknown |
| Reason for ETV failure | Free text entry |
| Date of Surgery | Any date/unknown |
| Patient Age at surgery | Formula=Date of surgery-Date of birth |
| Weight | Any weight in Kg |
| Suspected cause for hardware removal | Need to externalize (infection), need to externalize (pseudocyst), need to externalize (other), need to internalize (EVD removal), fractured shunt (proximal), fractured shunt (distal), obstruction (proximal), obstruction (distal), obstruction (valve), disconnection, over-drainage, reservoir malfunction, ventriculomegaly (not otherwise specified), truncated catheter, unknown, other |
| Imaging performed during admission | CT, MRI, both, none, unknown, other |
| Positive CSF culture during admission for shunt removal | Yes, no, unknown, other |
| Physician performing surgery | Free text (later coded into numbers) |
| Devices included under sample ID | Ventricular catheter, subdural catheter, subgaleal catheter, EVD, lumbar catheter, valve, peritoneal catheter, atrial catheter, intracranial pressure monitor, reservoir, CSF only, Other |
| Date of hardware insertion | Any date |
| Length of hardware insertion | Formula=date of surgery-date of insertion |
| What type (configuration) of shunt is this sample associate with? | VPS, VAS, LAS, LPS, other, N/A |
| (Ventricular catheters/EVDs only) Was the sample in contact with the ventricular walls or choroid plexus? | Yes (ventricular wall), yes (choroid plexus), yes (unknown), no, unknown, other, N/A |
| (Valves only) Valve setting | Free entry, any number |
| (Catheter/EVD only) Number of holes | Free entry, any integer |
| Electrocautery performed to free shunt? | Yes (bugbee/intraluminal), yes (electrocautery unspecified), no, unknown, N/A |
| Hardware brand | Codman (now Integra), Medtronic, Certas (now Integra), unknown, other |
| Surgical approach when failed hardware was inserted | Frontal, parietal, occipital, temporal, abdominal, subclavian, lumbar, other, N/A |
| Total number of revisions (prior to sample collection) | Free entry, any integer |
| Number of revisions without hardware change (prior to sample collection) | Free entry, any integer |
| Number of ventricular catheters (prior to sample collection) | Free entry, any integer |
| Number of revisions due to ventricular catheter obstruction (prior to sample collection) | Free entry, any integer |
| Number of peritoneal catheters (prior to sample collection) | Free entry, any integer |
| Number of atrial catheters (prior to sample collection) | Free entry, any integer |
| Number of lumbar catheters (prior to sample collection) | Free entry, any integer |
| Number of valves (prior to sample collection) | Free entry, any integer |
| Number of EVDs (prior to sample collection) | Free entry, any integer |
| Number of reservoirs (total, prior to sample collection) | Free entry, any integer |
| Number of reservoirs (ommaya only, prior to sample collection) | Free entry, any integer |
| Number of reservoirs (subgaleal only, prior to sample collection) | Free entry, any integer |
| Number of ICP monitors (prior to sample collection) | Free entry, any integer |
| History of other shunt configurations | Yes, No |
| If prior shunt configurations: number of prior configurations | 1, 2, 3, 4, 5, 6+ |
| For each prior configuration   1. Configuration type 2. Date configuration originally used 3. Date configuration was removed 4. Reason configuration was switched | 1. VPS, VAS, LPS, LAS, other 2. Any date/unknown 3. Any date/unknown 4. Free text entry |

*CNS* central nervous system *ETV* endoscopic third ventriculostomy *EVD* external ventricular drain *ICP* intracranial pressure *LAS* lumbo-atrial shunt *LPS* lumbo-peritoneal shunt *N/A* not applicable *VAS* ventriculo-atrial shunt *VPS* ventriculoperitoneal shunt
